# Neuropsychiatric symptoms of dementia and caregivers’ burden: a study among Indian caregivers

**DOI:** 10.1590/1980-5764-DN-2022-0017

**Published:** 2022-07-29

**Authors:** Ipsita Basu, Susmita Mukhopadhyay

**Affiliations:** 1 Indian Statistical Institute, Biological Anthropology Unit, Kolkata, West Bengal, India.

**Keywords:** Dementia, Neuropsychiatry, Caregivers, Tumor Burden, Demência, Neuropsiquiatria, Cuidadores, Carga Tumoral

## Abstract

Dementia is considered a most serious and disabling condition, affecting both the individual suffering from it and their caregiver.

**Objective:**

The study aimed to evaluate the relationship between neuropsychiatric problems of dementia and caregiver burden.

**Methods:**

A total of 138 caregivers of people with dementia participated in this cross-sectional study. The caregivers completed the questionnaires containing sociodemographic information as well as neuropsychiatric problems of dementia and caregiver burden.

**Results:**

The findings showed that all of the care-recipients were suffering from some kind of neuropsychiatric symptoms, the most common being apathy, anxiety, motor disturbance, and hallucination. Out of 12 symptoms, 11 were significantly associated with caregivers’ burden. The most important finding is that the severity of neuropsychiatric symptoms is highly responsible for severe caregivers’ burden.

**Conclusions:**

The identification of neuropsychiatric symptoms of dementia that influence caregiver burden is very critical for both caregivers’ and care-recipients’ health perspective. These findings can also be utilized to create care settings for demented people and help determine policies in the future.

## RESUMO

A demência é considerada a condição mais grave e incapacitante que afeta ao mesmo tempo tanto o indivíduo que a sofre como o seu cuidador.

**Objetivo:**

O estudo tem como objetivo avaliar a relação entre problemas neuropsiquiátricos de demência e sobrecarga do cuidador.

**Métodos:**

138 cuidadores de pessoas com demência participaram do estudo transversal. Os cuidadores preencheram os questionários contendo informações sociodemográficas, bem como problemas neuropsiquiátricos de demência e sobrecarga do cuidador.

**Resultados:**

Observou-se que todos os atendidos apresentavam algum tipo de sintoma neuropsiquiátrico; os mais comuns foram apatia, ansiedade, distúrbios motores e alucinações. Com exceção de um sintoma, 11 outros sintomas foram significativamente associados à sobrecarga dos cuidadores. O achado mais importante é que a gravidade dos sintomas neuropsiquiátricos é altamente responsável pela sobrecarga dos cuidadores.

**Conclusões:**

A identificação de sintomas neuropsiquiátricos de demência que influenciem a sobrecarga do cuidador é muito importante para a perspectiva de saúde dos cuidadores e dos receptores de cuidados. Essas descobertas também podem ser utilizadas para criar ambientes de atendimento para pessoas com demência e ajudar a determinar políticas no futuro.

## INTRODUCTION

The upswing in life expectancy and the aging of the population integrally favors the occurrence of many diseases in which age is a key factor, such as dementia. Dementia has been a global concern in recent years, as increase in elderly population, especially over 80 years old, would help escalate the number of dementia individuals in near future. Predictions indicated that the number of people living with dementia would rise from 47 million in 2015 to 75 million in 2030 and 135 million in 2050^1,2^. India is one of the countries that would be heavily impacted by dementia in near future. Moreover, while having the world’s second largest population, India has the fastest growing elderly population. It has previously been confirmed that dementia mostly affects the elderly and risk of having dementia doubles every 5 year for those older than 65 years and almost 40% for those aged 85 years and older^3^. In India, the number of people with dementia increases dramatically. According to a report, it has been predicted that by the year 2036, there would be 20,000–40,000 people living with dementia, even in states like West Bengal, India^4^.

Dementia is a gradual, widespread, and irreversible cognitive impairment that results in memory and other higher cognitive abilities loss. It is one of the conditions which severely impairs the capacity of an individual to carry out the activities of daily life, diminishing quality of life and autonomy of the individual who is experiencing dementia. It also causes changes in behavior and personality, which have a significant impact on the patient’s functional ability. As a result, the demented individual became dependent upon others with the progression of disease^5^.

The care of the demented person is usually provided by the family members. Therefore, the health of caregivers who care for demented people needs to be examined as caregiver burden might affect caregivers’ familial bonds, social relationships, and physical health, leading to psychological morbidity in caregivers and early institutionalization of patients. The majority of persons with dementia remain at home, and family members, mainly females (e.g., wife, daughter, sister, and daughter-in-law), are responsible for their daily care, which continues year after year^6^. Caregiving is physically and emotionally arduous as they have to provide their maximum time to their care-recipients and are unable to manage time for their leisure activities. Caregiver burden is a complex reaction to physical, psychological, emotional, social, and economic stressors connected with the caregiver’s care experience^7^. It was also reported that caregiver burden among main caregivers is an independent risk factor for higher death rates^8^.

A growing body of research compares between experience of dementia caregiving and caregiving for other types of dependence of family members. It was found that dementia caregivers experience tremendous burden compare to caregiving to the other types of dependence. Dementia caregiving is more time-consuming and had detrimental impact on caregivers’ emotional and social life as well. Caregivers also experienced deterioration of their mental as well as physical health at the same time^9–12^. Several studies have found that being a dementia caregiver causes psychological stress and mental health difficulties. It was also notable that caregivers’ health plays an essential influence on a patient’s institutionalization^13^. Many studies also reported that there are other several factors that may be linked with caregivers’ burden, such as age and gender of the caregiver, relationship with care-recipients, family history, types of work required, and duration of care hours and years^14–16^.

Indeed, literature has also revealed that the neuropsychological symptoms of dementia are prevalent and important issues that have immense impact on the quality of life of both patients and their caregivers. These symptoms do prevail throughout the course of dementia and are basically a wide range of psychological responses and typical behavior^17^. According to Finkel et al, neuropsychiatric symptoms are characterized as “symptoms of disturbed perception, thought content, mood or behaviour that frequently occur in patients with dementia”^18^. In contrast to cognitive symptoms, neuropsychiatric symptoms did not show a linear pattern of deterioration. Because of the unexpected and unruly nature of the neuropsychiatric symptoms, it is very difficult to manage. As a consequence of these symptoms, caregivers may experience higher levels of psychological health problems^19^. Several studies have found that early-stage symptoms of dementia and significant increases in symptoms are the predictors of caregiver burden over time^16,20^. A research found that wandering is the most prevalent symptom among people with dementia who experienced neuropsychiatric problems. It also has been linked to fall, injuries, and disorientation. Therefore, caregivers started worrying about the results of these incidents, which might increase their stress level^21,22^. It was found that among many other factors, these symptoms are closely associated with caregivers’ burden^23^.

In India, dementia is not considered as medical disorder that needs proper treatment in proper time, but rather a natural process of aging and remains as a hidden problem. Due to a lack of awareness of symptoms and progressive nature of dementia, people did not give serious attention to the condition. The challenges involved with dementia caregiving are still ignored, and gerontological research in India had not paid enough attention to them. Therefore, there is less Indian research evidence on dementia caregivers’ burden and its link to dementia-related neuropsychiatric problems. This study aimed to better understand (1) the neuropsychiatric symptoms of dementia present among a group of demented individual and (2) the link between neuropsychiatric problems and the burden experienced by their caregivers.

## METHODS

### Selection of study participants

This is a cross-sectional study carried out in West Bengal, India. The information of the caregivers was obtained from a nongovernmental organization. A total of 450 caregivers were contacted through phone and explained the purpose of this study. Out of this, 183 caregivers who were volunteered to participate were selected. The inclusion criteria were as follows:

1. Caregiver must be a primary family caregiver of a demented person;
2. Caregiver must be an adult;
3. Caregiver should have at least 1 year of experience providing care; and
4. Care-recipient must be clinically diagnosed with dementia.

Finally, a total of 138 caregivers who met the study criteria were recruited.

### Ethical clearance

The Institutional Review Board of Indian Statistical Institute in Kolkata reviewed the participant information document and the applicable informed consent form and provided an ethical clearance certificate. The majority of the participants were fluent in Bengali and English. However, a Bengali version of all instruments, fully translated by experts, was also provided to those who did not speak English well. To ensure authenticity, the same person answered each variation of the same question.

### Interview procedure

Researcher visited each and every residence as per caregivers’ convenience. The care-recipient’s medical report was initially reviewed. Then the study objectives and consent form were given to them. After signing the consent form, data collection procedure was started. Participants’ interview lasted for an hour, and selected questionnaires were given to them to fill up.

### Measures

Pre-tested questionnaire was developed to elicit the sociodemographic profile of caregivers and care-recipients. It included questions about the caregivers’ sex, age at the time of interview, education, occupation, marital status, relationship with care-recipients, family types, and monthly household expenditure [in Indian Rupees (INR)]. Information on care-recipient’s sex, age at the time of interview, education, type of dementia, and duration of suffering from dementia was also collected.

Dementia care-recipients’ neuropsychiatric symptoms, severity, and caregivers’ burden were assessed with the Neuropsychiatric Inventory (NPI)^24^. The NPI is a structured interview with a caregiver who is in close contact with people with dementia. It is evaluated based on 12 neuropsychiatric domains related to dementia, namely, delusions, hallucinations, agitation, dysphoria, anxiety, apathy, irritability, euphoria, disinhibition, aberrant motor behavior, nighttime behavior disturbances, and appetite and eating abnormalities. The caregivers were asked to fill in the questionnaire prepared on the basis of their experiences with the symptoms of the care-recipients. In case of the absence of any particular symptom of care-recipient, the subsequent query was skipped and moved to the next question. While in the presence of the abnormal behaviors of care-recipient, the behavioral domain is then explored with other sub-questions that provide more detailed information on that particular neuropsychiatric disturbance. In these sub-questions, the caregiver is asked to rate the frequency of the symptoms of that domain on a scale of 1–4 (1=occasionally, 2=once a week, 3=several time in a week, 4=very frequently) as well as their severity on a scale of 1–3 (1=mild, 2=moderate, 3=severe). Caregiver’s burden is rated on a 6-point scale, with 0=no burden, 1=minimal, 2=mild, 3=moderate, 4=severe, and 5=extreme. The total score for each domain is calculated by multiplying the frequency by the severity. A total score is calculated by adding all the domain scores. Severity of dementia was categories as mild, moderate, and severe. Similarly, caregiver burden score for each neuropsychiatric domain was obtained and a total burden score was calculated by adding all the 12 domains’ burden scores.

### Statistical analysis

Descriptive statistics were used to demonstrate the sociodemographic features of the caregivers and care-recipients as well as care-recipients’ neuropsychiatric problems. Chi-square test was performed to determine whether or not neuropsychiatric symptoms of dementia are associated with caregiver burden. A logistic regression analysis was carried out to evaluate the relationship between neuropsychiatric symptoms of dementia and caregiver burden to quantify the power of the relationship. A p-value of ≤0.05 was considered statistically significant for all inferential statistics. Data were analyzed using Power of Advanced Statistical Analysis version 18.0 (IBM Corp.).

## RESULTS

**Figure 1:**
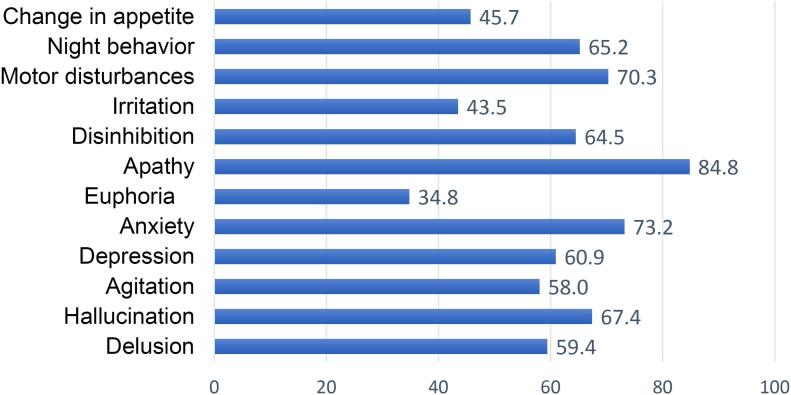
Graphical representation of care-recipients’ neuropsychiatric symptoms.

Sociodemographic characteristics of the caregivers and care-recipients are shown in Table 1. Most of the caregivers were above 55 years of age (68%), female (74%), and married (83%). The majority of the caregivers were graduate (82%), unemployed, and were mostly involved in household activities (68%). Spousal relationship (56%) was the most common form of relationship found between caregivers and care-recipients. About 53% had reported monthly household expenditure ranging between Rs. 26,000 and Rs. 50,000. In contrast, the number of people affected by dementia was same in each sex (i.e., 50% each for male and female). Mean age of the care-recipients was 75 years. Majority (58%) of the recipients were graduate. More than 55% of the care-recipients were suffering from dementia for less than 5 years. Alzheimer’s type of dementia was the most common type found among care-recipients, followed by vascular dementia. Figure 1 shows the neuropsychological symptoms of the care-recipients. The most prevalent symptom among the care-recipients was apathy (84.8%), followed by anxiety (73.2%), motor disturbances (70.3%), and hallucinations (67.4%).

**Table 1:** Information of caregivers and care-recipients.

Caregivers (n=138)			
Variables	Category	n	%
Gender	Male	35	25.4
	Female	103	74.6
Age group (in years)	<35	6	4.3
	36–55	37	26.8
	56+	95	68.8
	Mean age (years±sd)	61.35±13.86	
Marital status	Single	23	16.7
	Married	115	83.3
Education	Up to secondary	25	18.1
	Graduate	73	52.9
	Postgraduate and above	40	29.0
Occupation	Employed full time	10	7.3
	Employed part time	33	23.9
	No employment	95	68.8
Relationship with care-recipient	Wife	54	39.1
	Husband	24	17.4
	Daughter	26	18.8
	Son	8	5.8
	Others*	26	18.8
Monthly household expenditure (INR)	≤26,000	39	28.3
	26,001–50,000	74	53.6
	≥50,000	25	18.1
**Care-recipients (n=138)**			
Gender	Male	69	50.0
	Female	69	50.0
Age group (in years)	≤60	4	2.89
	61–70	31	22.46
	71–80	66	47.83
	>80	37	26.82
	Mean age (years±sd)	75.54±7.89	
Education	Up to secondary	21	15.2
	Graduate	80	58.0
	Postgraduate and above	37	26.8
Duration of suffering (in years)	≤5	76	55.1
	>5	62	44.9
Types of dementia	Alzheimer’s	104	75.37
	Vascular dementia	24	17.41
	Lewy body dementia	2	1.44
	Frontotemporal dementia	6	4.34
	Others	2	1.44

* Brother, sister, in-laws.

Table 2 shows the association between care-recipients’ neuropsychiatric symptoms and caregivers’ level of burden. It was observed that overall 60% of the caregivers experienced a severe level of burden. More than 50% of the caregivers who provided care to recipients with severe level of apathy experienced severe level of burden. In addition, more than 30% of the caregivers who cared for demented persons having severe level of hallucination, anxiety, motor disturbance, and night behavior experienced higher level of burden.

**Table 2:** Association between care-recipients’ neuropsychological symptoms and caregivers’ burden.

Symptoms	Categories	Level of burden		Chi-square	p-value
		Mild	Severe		
Delusion	Mild	35 (25.36)	37 (26.81)	30.823	<0.001*
	Moderate	3 (2.17)	19 (13.78)		
	Severe	2 (1.45)	42 (30.43)		
Hallucination	Mild	32 (23.19)	35 (25.36)	23.806	<0.001*
	Moderate	3 (2.17)	12 (8.70)		
	Severe	5 (3.62)	51 (36.96)		
Agitation	Mild	31 (22.46)	47 (34.06)	10.382	0.006*
	Moderate	3 (2.17)	24 (17.39)		
	Severe	6 (4.35)	27 (19.57)		
Depression	Mild	32 (23.19)	48 (34.78)	12.094	0.003*
	Moderate	4 (2.90)	16 (11.59)		
	Severe	4 (2.90)	34 (24.64)		
Anxiety	Mild	27 (19.57)	21 (15.22)	26.026	<0.001*
	Moderate	4 (2.90)	16 (11.59)		
	Severe	9 (6.52)	61 (44.20)		
Euphoria	Mild	77 (55.80)	33 (23.91)	0.952	0.658*
	Moderate	3 (2.17)	13 (9.42)		
	Severe	4 (2.90)	8 (5.80)		
Apathy	Mild	14 (10.14)	15 (10.87)	9.005	0.011
	Moderate	7 (5.07)	11 (7.97)		
	Severe	19 (13.78)	72 (52.17)		
Disinhibition	Mild	26 (18.84)	36 (26.09)	9.342	0.009
	Moderate	8 (5.80)	31 (22.46)		
	Severe	6 (4.35)	31 (22.46)		
Irritability	Mild	33 (23.91)	54 (39.14)	9.175	0.010*
	Moderate	2 (1.45)	11 (7.97)		
	Severe	5 (3.62)	33 (23.91)		
Motor disturbances	Mild	30 (21.74)	18 (13.04)	40.166	<0.001*
	Moderate	2 (1.45)	17 (12.32)		
	Severe	8 (5.80)	63 (45.65)		
Night behavior	Mild	31 (22.47)	33 (23.91)	22.047	<0.001*
	Moderate	3 (2.17)	17 (12.32)		
	Severe	6 (4.35)	48 (34.78)		
Change in appetite	Mild	35 (25.36)	54 (39.13)	13.249	<0.001*
	Moderate	2 (1.45)	25 (18.12)		
	Severe	3 (2.17)	19 (13.77)		

* Fisher’s exact test.

Table 3 shows the relationship between care-recipients’ neuropsychiatric symptoms and caregivers’ level of burden. It was observed that caregivers experienced severe level of burden while providing care for persons with serious neuropsychiatric symptoms. It was also shown that caregivers’ who provided care for persons with severe level of delusion, depression, anxiety, apathy, disinhibition, irritability, motor disturbances, and night behavior are likely to experience higher level of burden than caregivers who provided care to the person with moderate-to-mild level of neuropsychiatric symptoms.

**Table 3:** Result of binary logistic regression analysis between care-recipients’ neuropsychiatric symptoms and caregivers’ level of burden.

Symptoms	Category	B	SE	Sig	Exp(B)	95%CI	
						Lower	Upper
Delusion	Severe	2.989	0.761	<0.001	19.865	4.468	88.310
	Moderate	1.790	0.665	0.007	5.991	1.629	22.036
	Mild	Reference					
Hallucination	Severe	2.233	0.529	<0.001	9.326	3.309	26.281
	Moderate	1.297	0.690	0.060	3.657	0.945	14.148
	Mild	Reference					
Agitation	Severe	1.088	0.507	0.032	2.968	1.098	8.020
	Moderate	1.663	0.655	0.011	5.277	1.463	19.036
	Mild	Reference					
Depression	Severe	1.735	0.576	0.003	5.667	1.833	17.515
	Moderate	0.981	0.604	0.104	2.667	0.817	8.708
	Mild	Reference					
Anxiety	Severe	2.165	0.461	<0.001	8.714	3.533	21.493
	Moderate	1.638	0.630	0.009	5.143	1.495	17.686
	Mild	Reference					
Apathy	Severe	1.263	0.452	0.005	3.537	1.458	8.583
	Moderate	0.383	0.610	0.530	1.467	0.444	4.846
	Mild	Reference					
Disinhibition	Severe	1.317	0.515	0.011	3.731	1.360	10.238
	Moderate	1.029	0.473	0.029	2.799	1.108	7.069
	Mild	Reference					
Irritability	Severe	1.395	0.528	0.008	4.033	1.432	11.360
	Moderate	1.212	0.800	0.130	3.361	0.701	16.118
	Mild	Reference					
Motor disturbances	Severe	2.575	0.479	<0.001	13.125	5.130	33.582
	Moderate	2.651	0.805	<0.001	14.167	2.926	68.599
	Mild	Reference					
Night behavior	Severe	2.017	0.500	<0.001	7.515	2.820	20.026
	Moderate	1.672	0.679	0.013	5.323	1.420	19.960
	Mild	Reference					
Change in appetite	Severe	1.412	0.658	0.032	4.105	1.130	14.909
	Moderate	2.092	0.766	0.006	8.102	1.805	36.374
	Mild	Reference					
Constant		-4.764	1.214	<0.001	0.009		

Dependent variable: caregivers’ level of burden.

Table 4 shows the association between care-recipients’ neuropsychiatric problems and caregivers’ burden. It was found that caregivers (66.67%) who look after recipients with severe neuropsychiatric problems have higher level of burden. Significant association was found between care-recipients’ neuropsychiatric problems and caregivers’ level of burden.

**Table 4:** Association between care-recipients’ overall neuropsychiatric problem and caregivers’ burden.

Level of neuropsychiatric problem among care recipients	Caregivers’ burden due to neuropsychiatric problems among care recipients			
	Mild	Severe	Chi-square	p-value
Lower	28 (20.29)	6 (4.34)	62.424	<0.001
Higher	12 (8.70)	92 (66.67)		

Table 5 shows the relationship between care-recipients’ neuropsychiatric problems and caregivers’ burden. It was found that caregivers who provide care to recipients with severe neuropsychiatric problems experienced higher level of burden than those who provided care to recipients with mild neuropsychiatric problems. Significant relationship was found between care-recipients’ neuropsychiatric problems and caregivers’ burden.

**Table 5:** Result of logistic regression analysis using care-recipients’ overall neuropsychiatric problems and caregivers’ burden.

Dependent variable	Independent variable	Category	B	SE	Wald	Sig	Exp (B)	95%CI	
								Lower	Upper
Level of burden	Constant		2.037	0.307	44.042	<0.001	7.667		
	Neuropsychiatric problems among care-recipients	Severe	3.334	0.545	43.149	<0.001	1.028	0.081	10.233
		Mild	Reference group						

## DISCUSSION

The literature on the health of caregivers’ comes from all across the world. Noncommunicable disorders such as dementia are developing as a new health hazard as the population ages rapidly. As a result of the nature of dementia, persons suffering from it gradually lose their cognitive and functional capacities, becoming increasingly reliant on their family members for daily activities. However, studies on the neuropsychiatric issue of dementia and its impact on caregivers are rarely conducted in India. Therefore, a group of caregivers who provided care for a demented family member were chosen for this cross-sectional study to assess their degree of burden in relation to several neuropsychiatric issues associated with dementia.

Neuropsychiatric symptoms in dementia are more prevalent when dementia is well progressed. These symptoms are responsible for an individual’s effective functional impairment, dependence upon others, and increased caregiver burden. These issues can be present months or years before its actual diagnosis. These kind of symptoms have been observed to be more stressful to the caregivers than cognitive impairments^19^. This study observed that neuropsychiatric problems were present among all the care-recipients and it created burden on their caregivers. Overall, 12 neuropsychiatric problems were assessed, such as delusion, hallucination, agitation, depression, anxiety, euphoria, apathy, disinhibition, irritation, motor disturbance, night behavior, and change in appetite. It was found that apathy (84.8%) was the most common symptom found among care-recipients, followed by anxiety (73.2%), motor disturbances (70.3%), hallucination (67.4%), and night behavior (65.2%). A Brazilian study^25^ found that majority (91%) of the dementia individuals exhibit more than one neuropsychiatric symptoms, among which, agitation, aberrant motor behavior, and apathy were more prevalent. Various research studies evaluating the prevalence of neuropsychiatric symptoms in dementia patients have yielded varied results. The most prevalent symptoms in dementia patients were apathy, sadness, irritability, agitation, and anxiety, whereas euphoria, hallucinations, and disinhibition were the least common. The most major symptoms were apathy and anxiety, which also corroborate with our present study^26^.

Studies found that specific neuropsychiatric problems such as night behavior and agitation were more closely related to caregivers’ burden^27,28^. Our findings differ slightly from the previous study. Changes in outcomes may be attributable to differences in the characteristics of the participants group and the evaluation techniques. According to our findings, about 52% of caregivers had experienced higher degree of burden while caring for demented care-recipients with severe apathy. Aside from apathy, motor disturbance, night behavior, and anxiety all had a greater influence on caregiver burden. Like in other research, euphoria was the least predominant neuropsychiatric symptom along with less caregiver burden in our study^29^.

Hung et al made an observation among 88 dementia caregivers and found that caregivers’ burden increases with higher neuropsychiatric symptoms of recipients^30^. In another study involving 67 caregivers, Matsumoto et al found higher levels of distress in caregivers of patients with more neuropsychiatric symptoms. The results of a Brazilian study also reported that caregivers who provided care for individual with neuropsychiatric issues developed higher risk of depressive disorder, anxiety, insomnia, and related problems^31^. These findings are supported with our study findings. This study revealed that 67% of the caregivers experienced higher level of burden related to neuropsychiatric problems among care-recipients^32^. Most importantly, logistic regression analysis showed that caregivers who provided care to recipients with extreme neuropsychiatric disorders experienced significantly greater burden than those caregivers who gave care to recipients with less conspicuous neuropsychiatric disorders. It was also observed that caregivers’ poor mental health condition might result in low quality of life of care-recipients^33^. This study established a link between neuropsychiatric symptoms of care-recipients and caregivers’ burden and its impact on care-recipients’ quality of life^34^.

In India, caregivers’ health-related research, particularly for dementia caregivers, is extremely rare. As a result, this cross-sectional study may be regarded as a benchmark endeavor in caregivers’ health research in the Indian setting, particularly in terms of dementia caregivers in the eastern region. This study might in fact contribute to the current body of knowledge about caregivers’ health difficulties in India. There are some limitations as well. Due to cross-sectional nature of the study, researcher was not able to stay and observe the daily engagement of the caregivers toward their care recipients. The study is also limited to a particular ethnic group.

As the elderly population grows very fast, the demand of this area also increases with time. The identification of neuropsychiatric symptoms of dementia which influence caregiver burden is very crucial for healthy life for both caregiver and care-recipient. Overall, this study explained caregivers’ burden associated with neuropsychiatric symptoms. It was clearly evident from the study that caregivers’ burden was significantly associated with severity of care-recipients’ neuropsychiatric symptoms. Moreover, it was found that caregivers who provided care for person with severe neuropsychiatric issues experienced severe level of burden. These findings can also be used to design the care setting for demented individual and contribute to develop policy in future, thus adding very useful results to the growing body of research.
